# Biological Function and Mechanism of NAT10 in Cancer

**DOI:** 10.1002/cai2.154

**Published:** 2025-01-14

**Authors:** Yufeng Han, Xinxin Zhang, Lei Miao, Huiran Lin, Zhenjian Zhuo, Jing He, Wen Fu

**Affiliations:** ^1^ Guangdong Provincial Key Laboratory of Research in Structural Birth Defect Disease, Department of Pediatric Surgery, Guangzhou Women and Children's Medical Center, Guangzhou Institute of Pediatrics Guangzhou Medical University Guangzhou Guangdong China; ^2^ Faculty of Medicine Macau University of Science and Technology Macau China; ^3^ Laboratory Animal Center, School of Chemical Biology and Biotechnology Peking University Shenzhen Graduate School Shenzhen Guangdong China; ^4^ State Key Laboratory of Chemical Oncogenomics Peking University Shenzhen Graduate School Shenzhen Guangdong China

**Keywords:** acetyltransferase, cancer, N‐acetyltransferase 10, N4‐acetylcytidine

## Abstract

*N*‐acetyltransferase 10 (NAT10) is a nucleolar acetyltransferase with an acetylation catalytic function and can bind various protein and RNA molecules. As the N4‐acetylcytidine (ac4C) “writer” enzyme, NAT10 is reportedly involved in a variety of physiological and pathological activities. Currently, the NAT10‐related molecular mechanisms in various cancers are not fully understood. In this review, we first describe the cellular localization of NAT10 and then summarize its numerous biological functions. NAT10 is involved in various biological processes by mediating the acetylation of different proteins and RNAs. These biological functions are also associated with cancer progression and patient prognosis. We also review the mechanisms by which NAT10 plays roles in various cancer types. NAT10 can affect tumor cell proliferation, metastasis, and stress tolerance through its acetyltransferase properties. Further research into NAT10 functions and expression regulation in tumors will help explore its future potential in cancer diagnosis, treatment, and prognosis.

## Graphical abstract

*N*‐acetyltransferase 10 (NAT10) is an acetyltransferase that mediates the acetylation of proteins and RNA. NAT10 positively affects the proliferation, metastasis, invasion, and drug resistance of various cancers through the regulation of cancer‐related proteins and genes. NAT10 inhibitor has an improved effect on the development and drug resistance of cancers, and targeting NAT10 has potential value for cancer therapy.

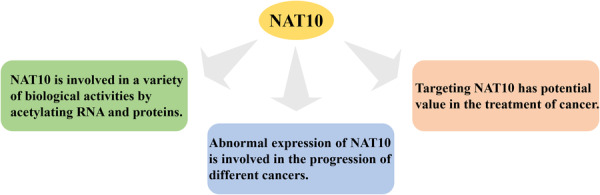

**Abbreviations**
ac4C: N4‐acetylcytidine
ADCY3: adenylate cyclase 3
AML: acute myeloid leukemia
BAX: Bcl‐2‐associated X protein
BC: breast cancer
Bik: BCL2‐interacting killer
BLCA: bladder urothelial carcinoma
BMP: bone morphogenetic protein
BMSC: bone marrow mesenchymal stem cell
CDS: coding sequence
CEP170: centrosomal protein 170
CRC: colorectal cancer
CRPC: castration‐resistant prostate cancer
DARS‐AS1: aspartyl‐tRNA synthetase antisense 1
DSB: DNA double‐strand break
EMT: epithelial–mesenchymal transition
ERS: endoplasmic reticulum stress
FA: fatty acid
FSP1: ferroptosis suppressor protein 1
GC: gastric cancer
HAAPIR: heart‐apoptosis‐associated piRNA
HAT: histone acetyltransferase
HCC: hepatocellular carcinoma
HGPS: Hutchinson‐Gilford progeria syndrome
HIF: hypoxia‐inducible factor
HNSCC: head and neck squamous cell carcinoma
Mdm2: Mouse double minute 2
MM: multiple myeloma
MORC2: MORC family CW‐type zinc finger 2
MSC: mesenchymal stem cell
NAT10: *N*‐acetyltransferase 10
NLS: nuclear localization signal
OGA: OGlcNAcase
OVX: ovariectomy
PARP1: Poly (ADP‐ribose) polymerase 1
PDAC: pancreatic ductal adenocarcinoma
PS‐ASO: phosphorothioate oligonucleotide
ROS: reactive oxygen species
RUNX2: Runt‐related transcription factor 2
siRNA: small interfering RNA
SLE: systemic lupus erythematosus
snoRNA: small nucleolar RNA
TBL3: transducin beta‐like protein 3
Tfec: transcription factor EC
THUMPD1: THUMP domain protein 1
TME: tumor microenvironment
UBF: upstream binding factor
ULK1: UNC‐52‐like kinase 1
UPR: unfolded protein response

## 1 Introduction

*N*‐acetyltransferase 10 (NAT10, previously called hALP) is a histone acetyltransferase (HAT) and a member of the GNAT protein family responsible for the acetylation of lysine residues. It is also the only known enzyme that mediates N4‐acetylcytidine (ac4C) modifications in eukaryotic RNA, known as the ac4C “writer.” NAT10 is a nucleolar protein composed of 1025 amino acids with a molecular weight of 116 kDa. Its gene is located on chromosome 11p13, with the DNA sequence extending from 34,105,617 to 34,147,670, and encodes nine transcripts. According to InterPro database predictions, the NAT10 structure may include a GNAT domain, helicase domain, Acyl‐CoA N‐acyltransferase, tRNA^Met^ cytidine acetyltransferase TmcA, and P‐loop NTPase fold. The GNAT domain is involved in transferring the acetyl group of acetyl‐CoA and therefore being the most important portion of NAT10 for its acetyltransferase function [1]. The RNA helicase and acetyltransferase domains are the relevant regions for the NAT10 RNA acetylation modification function [2, 3]. TmcA ensures the AUG codons are accurately identified by catalyzing ac4C modifications of the tRNA^Met^ wobble position [4]. The P‐loop NTPase fold can hydrolyze the ATP/GTP β‐γ phosphate bond to provide energy for the acetylation modification [5].

NAT10 can bind and acetylate both proteins and RNAs, thereby participating in a diverse range of biological activities by targeting different molecules. As previously described, NAT10 has acetyltransferase activity targeting the α‐tubulin [6], histone [7], and p53 [8] proteins. In 2003, NAT10 was first reported to have histone acetylation activity and enhance telomerase activity by upregulating hTERT expression levels [7]. Subsequent studies have shown that NAT10 plays important roles in DNA damage repair [9], cell division [6], and ribosome synthesis [10] by targeting various proteins and RNAs. Additionally, NAT10 can maintain the high heat resistance of cells [11] and improve the fidelity of protein translation [12] through tRNA ac4C modifications. It can also mediate ribosomal RNA (rRNA) ac4C modifications, which contribute to accurate protein translation [13]. NAT10‐mediated ac4C modifications of tRNA and rRNA molecules reportedly require the assistance of the respective cofactors THUMP domain protein 1 (THUMPD1) and small nucleolar RNA (snoRNA) [13]. Recent studies have also revealed that NAT10 can mediate ac4C modifications of mRNA molecules, which enhance mRNA stability and translation efficiency [14]. This function has implications for the development of novel therapeutic intervention methods for diseases such as cancer. However, it is currently unknown whether a cofactor is required for NAT10‐mediated ac4C modification of mRNAs.

Much of the recent research on NAT10 has focused on its effects on different diseases, particularly various cancers. Larrieu et al. first found that inhibiting NAT10 activity could improve nuclear shape in laminopathic cells and thus is suitable for treating Hutchinson‐Gilford progeria syndrome (HGPS) [15]. High NAT10 expression levels have been observed in hepatocellular carcinoma (HCC) tissues compared with normal tissues, with increased NAT10 expression patterns predicting lower patient survival rates [16]. NAT10 is also associated with other malignant diseases, such as breast cancer (BC) [17], gastric cancer (GC) [18], and colorectal cancer (CRC) [19]. NAT10 has been shown to enhance the tumor cell proliferation, metastasis, and invasion abilities of various cancers, resulting in adverse consequences for patient treatment and prognosis [20].

Here, we provide a systematic review of recent studies describing the various biological mechanisms of NAT10 and its roles in different cancers. Moreover, we discuss the possibility of NAT10 as a target for disease treatment.

## 2 NAT10 Cellular Localization

The NAT10 protein in cells is localized to the nucleolus, which is determined by the nuclear localization signal (NLS) [21]. After DNA damage occurs or cancer develops, NAT10 can translocate. For example, it is transferred to the nucleoplasm and activates p53 via acetylation in response to DNA damage [8]. NAT10 can also translocate to the cytoplasm to promote ɑ‐tubulin acetylation and enhance microtubule stability, further affecting cancer cell migration and invasion [22]. Studies have shown that the two NAT10 NLSs, which include residues 68–75 and 989–1018, jointly determine its nuclear localization [22]. For instance, deleting residues 989–1018 results in NAT10 translocation from the nucleolus to the cytoplasm, while further deletion of residues 68–75 leads to NAT10 completely transferring to the cytoplasm and cell membrane. Interestingly, removing only residues 68–75 does not result in NAT10 translocation. Moreover, residues 549–834 have also been shown to be critical for NAT10 subcellular localization in the nucleolus or midbody, with the absence of these residues leading to cytoplasmic diffusion [6].

## 3 NAT10 Biological Functions

**Figure 1:**
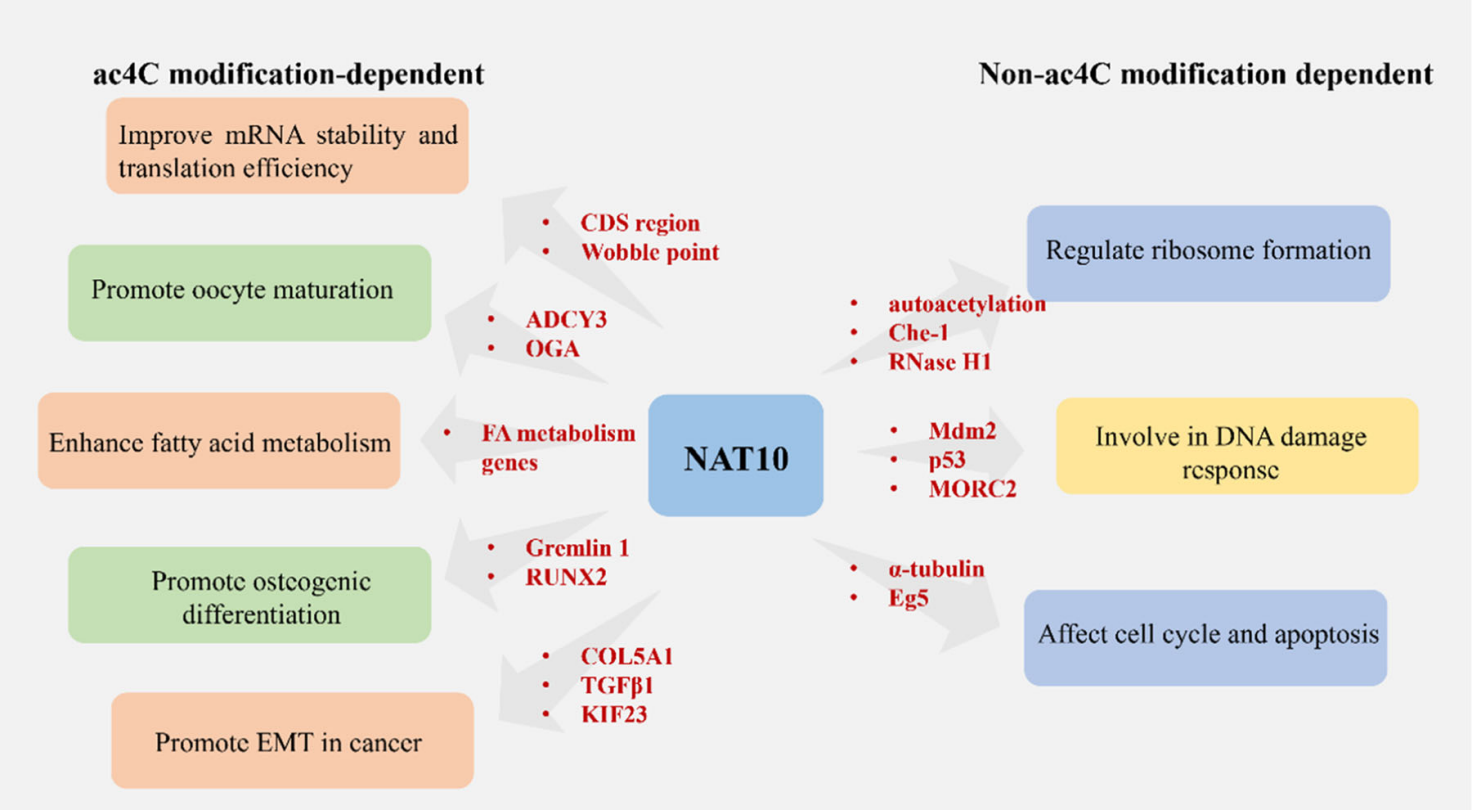
The biological functions of NAT10. NAT10 participates in biological processes in both ac4C modification‐dependent and non‐ac4C modification‐dependent manners. ac4C, N4‐acetylcytidine; NAT10, *N*‐acetyltransferase 10.

NAT10 has diverse biological functions that can be categorized as ac4C modification‐dependent or non‐ac4C modification‐dependent (Figure 1). NAT10 is involved in regulating mRNA stability and translation efficiency, osteogenesis, oocyte maturation, fatty acid (FA) metabolism, and the epithelial–mesenchymal transition (EMT) in an ac4C modification‐dependent manner [23]. NAT10 also plays an important role in ribosome formation, the DNA damage response, cell cycle, and apoptosis by mediating the acetylation of protein and RNA molecules. These biological functions are closely related to the occurrence, development, prognosis, and treatment of cancer.

### 3.1 Ac4C‐Dependent Biological Functions of NAT10

#### 3.1.1 Promotion of mRNA Stability and Translation Efficiency

**Figure 2:**
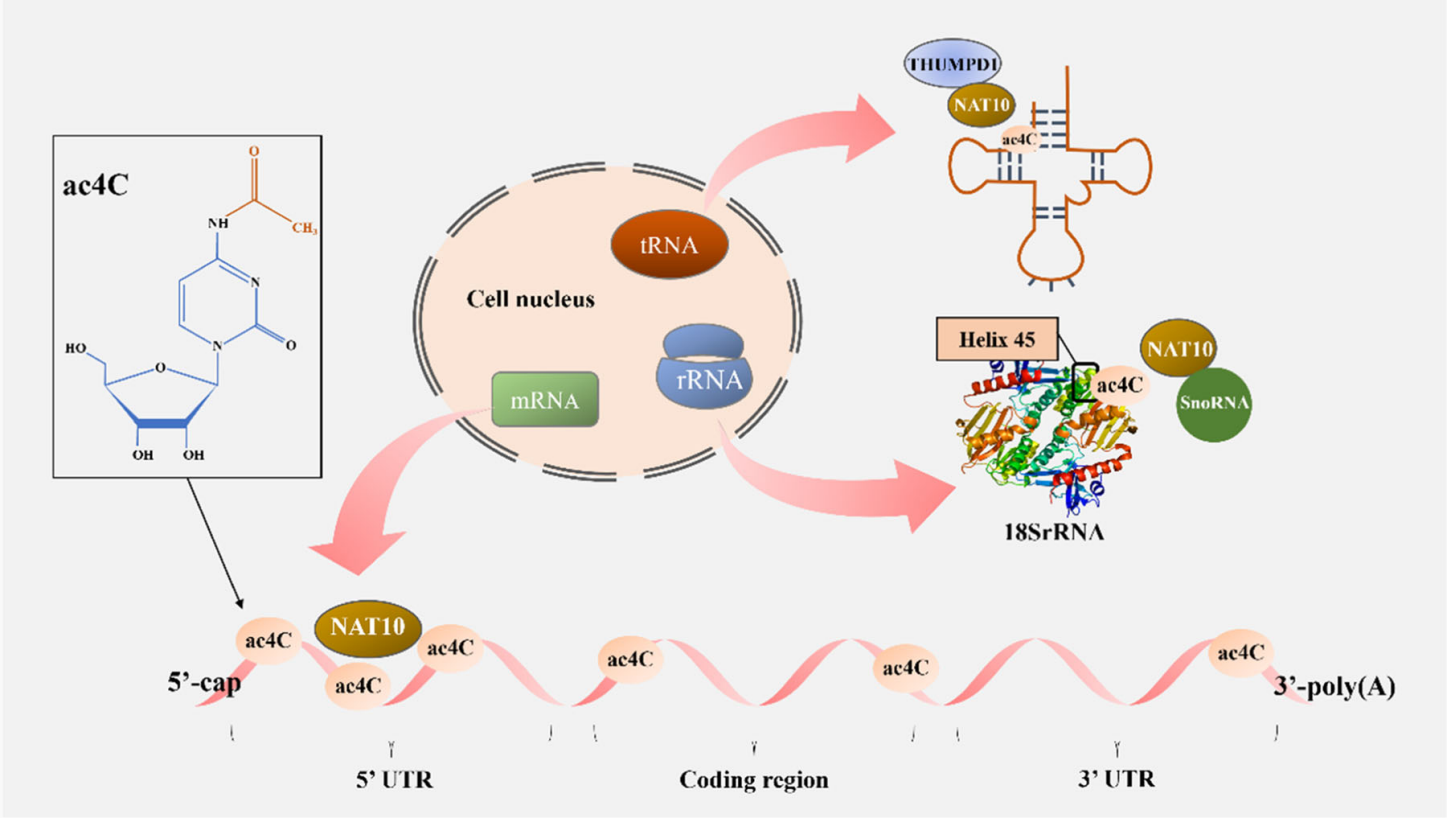
NAT10 mediates the ac4C modification of mRNAs, 18SrRNA, and tRNAs. NAT10 mediates tRNA ac4C modifications with the help of cofactor THUMPD1. NAT10 requires short nucleolar RNA (snoRNA) for the ac4C modification of site 1842 in helix 45 of human 18SrRNA. NAT10‐mediated mRNA ac4C modifications decrease from the 5' cap to the 3' end. ac4C, N4‐acetylcytidine; NAT10, *N*‐acetyltransferase 10.

Localization analysis has shown that NAT10‐mediated mRNA ac4C modifications are widespread throughout the human transcriptome. These are mainly concentrated in the coding sequence (CDS) region, decreasing gradually from the 5' end to the 3' end along the transcript (Figure 2). When NAT10 is absent, ac4C modifications in the CDS region will be decreased, resulting in a significantly shortened mRNA half‐life that affects its stability. When analyzing the ability of XRN‐1 (a 5' to 3' exonuclease) to degrade an in vitro‐transcribed radiolabeled reporter gene in the presence or absence of ac4C, no difference in XRN‐1 activity was observed between the two conditions. The results showed that ac4C could improve mRNA stability by uncoupling it against exonuclease resistance [14]. Subsequent studies further showed that NAT10 protects mRNA molecules from nucleic acid exonuclease attack by recruiting HNRNPQ and THRAP3 [3]. Knockdown of HNRNPQ or THRAP3 significantly decreased the expression levels of NAT10‐targeted RNAs, suggesting that NAT10‐mediated mRNA regulation is dependent on the involvement of HNRNPQ and THRAP3.

Generally, mRNA stability affects translation efficiency, with reduced mRNA stability leading to decreased translation [24]. In addition to affecting translation efficiency by improving mRNA stability, NAT10 may facilitate translation via other mechanisms. The ac4C modification of the tRNA^met^ wobble site prevents Watson–Crick base‐pairing site shielding and contributes to the correct decoding of methionine in bacteria [12]. As supported by tRNA acetylation in *E. coli*, mRNA acetylation in human cells should improve tRNA recognition to promote decoding efficiency. Consistent with this, ac4C at the wobble point contributes to mRNA translation. High cytidine concentrations within the wobble point have been observed in the ac4C peak, suggesting that ac4C directly acts on the ribosome decoding process and improves translation efficiency [14]. However, recent research has found that ac4C modifications in the Kozak sequence of AUG‐flanking can inhibit translation initiation and efficiency [25]. Similarly, NAT10‐mediated ac4C modification was found to accelerate *Gremlin 1* mRNA degradation, possibly by targeting its 3′ untranslated region (UTR) sites [26]. These findings indicate that the effects of ac4C modifications on mRNA stability and translation may be location‐dependent.

#### 3.1.2 Positive Effects on Osteogenic Differentiation

Mesenchymal stem cells (MSCs), which have osteogenic differentiation abilities, play an important role in regulating bone metabolic homeostasis in vivo [27]. Gremlin 1 is a bone morphogenetic protein (BMP) antagonist that targets BMP2 and BMP4. BMPs can activate the Smad1/5/9 signaling pathways, which play important roles in MSC osteogenesis [28]. Recent studies have shown that NAT10 can positively regulate the MSC osteogenic differentiation ability by increasing the ac4C levels of *Gremlin 1* mRNA, suggesting that NAT10 is an anti‐osteoporotic factor. NAT10 accelerates the *Gremlin 1* mRNA degradation rate by mediating its ac4C modification, which activates the BMP/Smad signaling pathway to promote MSC osteogenic differentiation [26]. Notably, as mentioned above, ac4C modification generally improves mRNA stability, implying that the effects of NAT10 on mRNAs may vary depending on the specific cell type or gene.

In addition, low NAT10 expression levels were observed in ovariectomy (OVX) mice and patients with osteoporosis. However, overexpression of NAT10 reversed bone loss in OVX mice. Furthermore, bone marrow mesenchymal stem cells (BMSCs) could form fewer bone nodules in vitro when NAT10 expression was silenced, while more bone nodules formed following NAT10 overexpression. Runt‐related transcription factor 2 (RUNX2) is a transcription factor that determines BMSC osteogenic differentiation. NAT10 can prolong the half‐life of *RUNX2* mRNA, thereby increasing *RUNX2* expression levels, by mediating ac4C modification. Therefore, NAT10 can regulate BMSC osteogenic differentiation through ac4C modification of *RUNX2* mRNA [29]. According to the abovementioned studies, NAT10 can regulate bone metabolism and prevent bone loss, making this protein a potential therapeutic target for treating osteoporosis.

#### 3.1.3 Regulation of Oocyte Maturation

One report described how the mRNA stability and translational regulation of oocyte maturation‐related genes are important factors that affect oocyte meiotic maturation [30]. Adenylate cyclase 3 (ADCY3) catalyzes ATP cyclization into cAMP, which is critical for maintaining oocyte meiosis stagnation [31]. NAT10 can mediate the ac4C modification of *ADCY3* mRNA, with ADCY3 protein expression levels being downregulated along with NAT10 during oocyte maturation [32]. Downregulation of NAT10 leads to ADCY3 degradation, resulting in reduced cAMP levels that ultimately stimulate the recovery of meiosis. However, when NAT10 expression was knocked down using small interfering RNAs (siRNAs) in GV‐stage oocytes, ac4C modification levels were reduced. This resulted in a significant decrease in the extrusion rate of the first body and a significant delay in oocyte meiosis maturation in vitro. This may have been caused by the abnormal reduction of ADCY3 and cAMP levels following NAT10 knockdown, resulting in signaling pathway crosstalk that thereby impaired meiosis. In addition, Transducin beta‐like protein 3 (TBL3) is considered a potential ac4C reader that regulates the downstream biological activity of ac4C modifications by directly or indirectly binding to ac4C‐modified RNAs [32]. However, it has not been confirmed if TBL3 can mediate the function of ac4C in oocyte maturation.

Furthermore, NAT10 can affect oocyte maturation by mediating the ac4C modification of its target gene OGlcNAcase (*OGA*). OGA is an enzyme that removes O‐GlcNAc modifications from its target proteins [33]. *OGA* mRNA expression levels increase with oocyte maturation, while O‐GlcNAc modification levels decrease. OGA plays a crucial role in oocyte maturation by mediating the O‐GlcNAc modification of proteins. With decreased OGA expression levels, oocyte meiosis maturation was hindered with increased O‐GlcNAc levels. Therefore, NAT10 may influence the levels of O‐GlcNAc‐modified proteins by regulating *OGA* expression, thus regulating oocyte maturation [34].

#### 3.1.4 Promotion of FA Metabolism

NAT10 is a key factor that affects FA metabolism and lipid accumulation in an ac4C modification‐dependent manner [35]. Inhibition of NAT10 activity may lead to decreased expression patterns of mitochondrial lipid metabolism enzymes. Reduced levels of MECR and ECHS1 have been observed in cells following NAT10 knockdown. MECR and ECHS1 participate in mitochondrial FA elongation and are essential for mitochondrial lipid metabolism [36]. Lipids are known to be essential for cell survival and proliferation. Depleting NAT10 could decrease the expression levels of FA metabolism genes, such as *ACSL1*, *ACSL3*, *ACSL4*, *ACAT1*, *ACDSB*, and *ELOVL6*. After NAT10 was depleted in cancer cells, lipid levels were reduced and FA metabolism was blocked, which led to cell death [35]. NAT10‐mediated ac4C modification can facilitate FA metabolism in cancer cells by improving the stability of FA metabolism genes. Therefore, targeting NAT10 has potential value for cancer treatment.

#### 3.1.5 Promotion of the EMT in Cancer

The EMT can accelerate tumor metastasis, thus stimulating tumor development and progression [37]. NAT10 can regulate the Wnt/β‐catenin signaling pathway in CRC cells through *KIF23* mRNA ac4C modification [19]. The Wnt/β‐catenin signaling pathways are often reported to play key roles in the EMT [37, 38, 39], with activation of these pathways facilitating the EMT process. Moreover, NAT10 can maintain *TGFβ1* mRNA stability and its protein levels through ac4C modification, thereby accelerating the EMT [40]. NAT10 can also mediate *COL5A1* mRNA ac4C modification. COL5A1 is a marker of EMT Ⅱ that can directly promote the EMT [18]. In HCC, downregulated NAT10 expression levels are often accompanied by increased E‐cadherin and decreased vimentin expression patterns, both of which are EMT markers [41]. However, NAT10 levels are positively correlated with E‐cadherin levels in CRC [42], potentially suggesting a discrepant regulatory effect of NAT10 on E‐cadherin in different tumor types.

### 3.2 Non‐ac4C‐Dependent Biological Functions of NAT10

#### 3.2.1 NAT10 is Involved in Ribosome Formation

The synthesis and posttranscriptional modification of rRNA are essential for ribosome biogenesis. NAT10 can activate RNA polymerase I transcription by binding to upstream binding factor (UBF) [43]. Pre‐rRNA transcription by RNA polymerase I in the nucleolus is the initial step of ribosome construction [21]. Moreover, the autoacetylation of NAT10 at K426 is required to enable its activation of rRNA transcription. The K426 mutation cannot acetylate UBF after binding to it, resulting in the reduced recruitment of RAF53 and RNA polymerase Ⅰ to rRNA [44]. When NAT10 is deacetylated by Sirt1, NAT10‐mediated activation of rRNA synthesis is inhibited. The NAT10‐mediated inhibition of Che‐1 transcription via autophagy regulator Che‐1 K228 acetylation is then eliminated. This suggests that NAT10 deacetylation under energy stress leads to a shift from rRNA synthesis to autophagy [45]. In addition, NAT10 can facilitate the function of RNase H1 in R‐loop solving, promoting RNase H1 endoribonuclease activity and enhancing phosphorothioate oligonucleotide (PS‐ASO) activity in cells. Reduced NAT10 levels will therefore decrease the unwinding of the R‐loop and increase PS‐ASO‐related cytotoxicity, thus damaging pre‐rRNA processing [46]. Toxic PS‐ASO can alter NAT10 protein localization, which will affect NAT10 functions.

Notably, research has indicated that NAT10 is also involved in regulating ribosome formation by mediating ac4C modifications. NAT10 mediates ac4C modification of the 1842 site in the human 18SrRNA helix 45 to participate in 18SrRNA formation during pre‐rRNA processing (Figure 2) [10]. The vertebrate‐specific box C/D snoRNA SNORD13 was found to contribute to 18S rRNA acetylation [13, 47]. As mentioned above, NAT10 can also catalyze the ac4C modification of mRNA and tRNA molecules, and the impact of these processes on ribosome synthesis is noteworthy.

#### 3.2.2 NAT10 Affects the DNA Damage Response

Studies have shown that NAT10 is mainly localized in the nucleus in the presence of genotoxic agents. The NAT10 gene promoter is activated to upregulate its expression, which in turn can enhance the resistance of cells to DNA damage [9]. NAT10 can enhance cell resistance to DNA damage by acetylating the lysine 767 site of MORC family CW‐type zinc finger 2 (MORC2). Under normal conditions, this modification is counteracted by the deacetylase SIRT2 [17]. Poly (ADP‐ribose) polymerase 1 (PARP1) can mediate NAT10 PARylation during DNA damage, which contributes to NAT10 translocation to the nucleoplasm and controls its interaction with MORC2 [48].

As a genome guardian, p53 has multiple key roles in biological processes, including in DNA damage repair, cell cycle arrest, and apoptosis. Studies have shown that NAT10 is involved in the regulation of p53. In the normal state, NAT10 serves as an E3 ligase of Mouse double minute 2 (Mdm2). Ubiquitinating Mdm2 promotes its degradation, thereby inhibiting the ubiquitination of p53. The NAT10 E3 ligase activity involves residues 456–558. Following DNA damage, NAT10 translocates to the nucleoplasm and acetylates p53 at K120 to reduce its ubiquitination level, thus supporting p53 activation [8]. Moreover, NAT10 can regulate telomerase activity by promoting the transcription of hTERT, which is closely related to the DNA damage response [7].

#### 3.2.3 NAT10 Regulates the Cell Cycle and Apoptosis

NAT10 protein localization varies with the progression of the cell division process. During late mitosis, NAT10 primarily accumulates within the midbody and increases α‐tubulin acetylation in this region. The absence of NAT10 can affect the recombination of nucleolus, as well as the stability of the midbody and tubulin in telophase, resulting in G2/M cell cycle arrest [6]. NAT10 can acetylate Eg5 at K771, which increases its stability. Eg5 is a motor protein that is important for spindle bipolarity [49]. NAT10 depletion can reduce Eg5 levels, resulting in impaired centrosome poleward motion and asymmetric spindle formation, eventually leading to mitosis catastrophe [50]. Moreover, NAT10 can enhance endoplasmic reticulum stress (ERS) and inhibit acute myeloid leukemia (AML) cell apoptosis. Research has indicated that the mechanism of NAT10 inhibition‐mediated cell cycle arrest upregulated p16 and p21 tumor suppressor protein expression and downregulated cell cycle checkpoint protein expression [51]. NAT10 can also regulate the cell cycle and apoptosis in a p53‐dependent manner [8].

In addition, NAT10 can regulate the ac4C modification of meiotic prophase I functional genes. Loss of NAT10 leads to dysregulated transcription, thus resulting in meiosis entry arrest that affects homologous recombination and DNA double‐strand break (DSB) repair [52]. Heart‐apoptosis‐associated piRNA (HAAPIR) was found to modulate cardiomyocyte apoptosis after myocardial infarction by targeting NAT10. HAAPIR promotes NAT10‐mediated ac4C modification of transcription factor EC (Tfec) mRNA and upregulates Tfec expression levels, further inducing BCL2‐interacting killer (Bik) transcription that ultimately leads to Bik accumulation and cardiomyocyte apoptosis [53]. Moreover, high NAT10 expression levels can inhibit multiple myeloma (MM) cell apoptosis by mediating *BCL‐XL* mRNA ac4C modification [54]. Therefore, NAT10 plays crucial roles in various pathways that regulate the cell cycle and apoptosis through both ac4C modification‐dependent and non‐ac4C modification‐dependent mechanisms.

### 3.3 The Effects of NAT10 Biological Functions on Cancer

**Figure 3:**
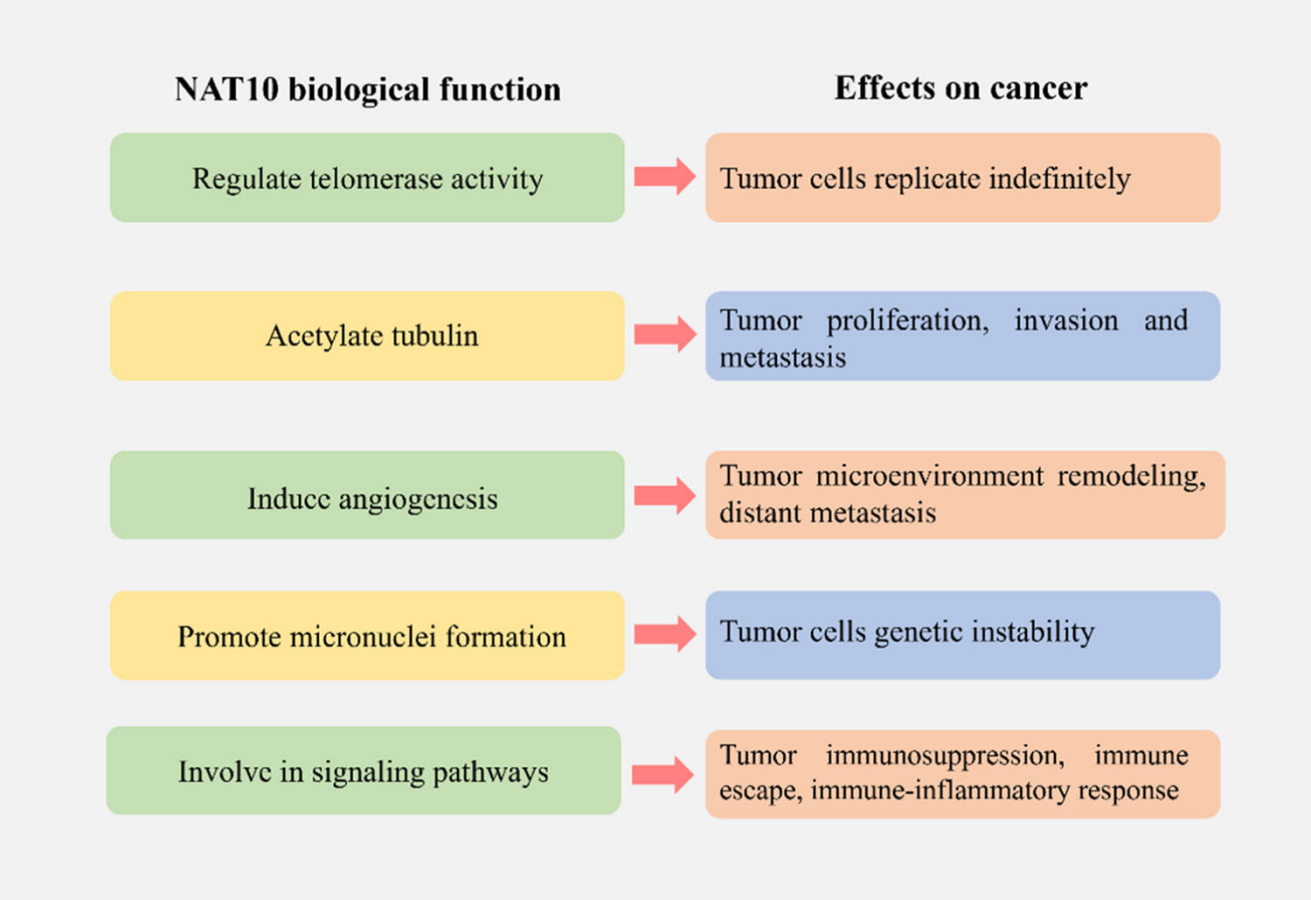
Effects of the NAT10 biological functions on cancer. NAT10, *N*‐acetyltransferase 10.

NAT10 biological functions are often involved in the occurrence and progression of cancer (Figure 3). NAT10 can regulate telomerase activity, specifically enhancing it by promoting hTERT expression [7]. Cancer cells typically have elevated telomerase activity compared with normal cells, which contributes to their unrestricted growth and division. However, subsequent studies unexpectedly found that overexpressing NAT10 shortened telomere length, which is generally unfavorable for cancer cells [55]. Because this contradicted previous predictions, a more complex relationship may exist between NAT10 and telomerase activity in tumors. NAT10 has been shown to increase tubulin protein stability through acetylation, which in turn impacts tumor invasion and metastasis [22]. In addition, stable microtubules can enhance mitosis levels and promote cancer cell proliferation [6].

NAT10 can induce angiogenesis, as it has been found to promote the transcription of the genes encoding CXCL3 and CXCL8. These cytokines can promote angiogenesis in tumors, as well as support immune cell recruitment and tumor microenvironment (TME) remodeling [56]. NAT10 can also affect angiogenesis through the TGF‐β pathway, thereby promoting distant metastasis of tumors [57].

NAT10 is involved in the regulation of DNA replication and DNA damage stress, which can promote micronuclei formation. Research has shown that inhibiting NAT10 can restrict DNA replication during mitosis, thus reducing the formation of micronuclei. However, the mechanism by which NAT10 promotes this process has not been determined. Micronuclei are often observed in cancer cells, with their formation being closely related to DNA damage and chromosomal malformation. The presence of micronuclei in cancer cells usually indicates genetic instability [58, 59].

NAT10 is involved in the regulation of multiple signaling pathways, including activating several pathways that affect immunity and inflammation by regulating cytokine levels. Pathway analysis has shown that NAT10 is significantly associated with TGF‐β signaling, NF‐κB activation signaling, reactive oxygen species (ROS)‐induced cellular signaling, and other pathways [60]. The TGF‐β signaling pathway plays a key role in cancer cell immunosuppression and immune evasion. Interfering with this pathway can contribute to the development of inflammatory diseases [61]. NAT10 can promote ROS production by increasing *Nox2* mRNA stability and expression levels, with the excessive ROS enhancing the LPS‐induced inflammatory response of macrophages through the NF‐κB signaling pathway [62]. NAT10 can modify key genes that mediate immune and inflammatory responses, such as *USP18*, *GPX1*, and *RGL1*. These genes influence autoimmune disease pathogenesis by regulating immune and inflammatory responses, as well as cytokine‐mediated signaling pathways [60]. In sepsis, NAT10 downregulation leads to decreased ULK1 expression levels and activation of the STING‐IRF3 signaling pathway, subsequently causing elevation of the pyroptosis‐inducing NLRP3 inflammasome in neutrophils [63]. These studies collectively demonstrate that the effects of NAT10 on cancer are complex and varied. Therefore, further studies are required to validate additional mechanisms and clarify the role of NAT10 in different cancer types.

## 4 The Role of NAT10 in Cancer

Published data have demonstrated that NAT10 can acetylate certain downstream targets, affect the EMT, and regulate the cell cycle and apoptosis. These events then affect tumor cell proliferation, metastasis, invasion, and drug resistance (Table 1).

**Table 1:** The NAT10 mechanisms of action in various human cancers.

Cancer type	Targets	Action effect on tumor
HCC	Mdm2/p53	Counteract the effect of Mdm2 on p53 and promote the proliferation of cells carrying p53 mutations
	ɑ‐tubulin	Increase migration and invasion
	HSP90AA1	Promote metastasis, antiapoptotic, and lenvatinib resistance
GC	COL5A1	Promote cell migration and EMT
	DARS‐AS1/miR‐330‐3p	DARS‐AS1 upregulates NAT10 by sponging miR‐330‐3p, promoting the proliferation, migration, and invasion
	MDM2	Promote the occurrence and progression of GC
	SEPT9/HIF‐1α	Enhance the hypoxia tolerance of GC cells
CRC	FSP1	Inhibit ferroptosis and promote proliferation, migration, and invasion
	miR‐6716‐5p/E‐cadherin	miR‐6716‐5p reduces E‐cadherin by inhibiting NAT10, promoting the EMT, motility, and invasion
BLCA	BCL9L/SOX4/AKT1	Promote the progression of bladder urothelial carcinoma
	AHNAK	Promote the cisplatin chemoresistance of cells
BC	MORC2	Regulate G2 checkpoint and enhance the sensitivity of the cell to DNA‐damaging agents
	AKT‐GSK3β pathway	Promote migration and invasion of cells
PDAC	LINC00623/USP39	LINC00623 maintains the stability of NAT10 by facilitating the interaction between NAT10 and USP39
	AXL	Promote the progression
	TGF‐β pathway	Contribute to distal metastasis
MM	BCL‐XL	Inhibit apoptosis and promote tumor proliferation
	PI3K‐AKT pathway	Inhibit apoptosis and promote tumor proliferation
	CEP170	Promote cell proliferation and chromosomal instability
AML	cyclins/p16/p21	Downregulation of NAT10 reduces cyclin and increases p16 and p21, inducing apoptosis
	Bax/Bak/Bcl‐2	Inhibition of NAT0 can increase Bax and Bak, decrease Bcl‐2, and activate the classical apoptotic pathway
	UPR pathway	Targeting NAT10 activates the UPR pathway, leading to apoptosis

Abbreviations: AML, acute myeloid leukemia; BC, breast cancer; BLCA, bladder urothelial carcin oma; CRC, colorectal cancer; EMT, epithelial–mesenchymal transition; GC, Gastric cancer; HCC, hepatocellular carcinoma; MM, multiple myeloma; PDAC, pancreatic ductal adenocarcinoma.

### 4.1 HCC

NAT10 expression levels are upregulated and localized in the cytoplasm in HCC cells, which counteracts the ubiquitination effect of Mdm2 on mutant p53. This results in increased levels of mutant p53, further promoting the proliferation of cells expressing this mutant and ultimately leading to adverse consequences for patients [16]. In addition, NAT10 translocation into the cytoplasm and cell membrane promotes ɑ‐tubulin acetylation and increases microtubule stability, thus leading to higher HCC migration and invasion rates [22]. NAT10 also contributes to improving HCC cell resistance to doxorubicin by facilitating the EMT [64]. It mediates the ac4C modification of *HSP90AA1* mRNA to upregulate *HSP90AA1* expression levels, thus promoting HCC metastasis, antiapoptotic ability, and lenvatinib resistance [65].

*COL15A*, *G6PD*, and *TP5313* mRNAs, which can be modified by NAT10‐mediated ac4C, were selected to construct a risk model. Bioinformatics analysis demonstrated that the model was suitable for predicting stem cells, TME distribution, and survival outcomes in HCC patients, as well as in patients with lung cancer and pancreatic cancer [66].

### 4.2 GC

NAT10 overexpression can promote the migration and EMT process of GC cells. Additionally, high NAT10 expression levels are significantly associated with poor GC patient survival rates. The effects of NAT10 on GC cells may be related to COL5A1 [18]. NAT10 mediates *COL5A1* mRNA ac4C modifications, which help maintain *COL5A1* mRNA stability without affecting COL5A1 protein activity. However, a subsequent report challenged this perspective by indicating that NAT10 neither modifies *COL5A1* mRNA nor regulates its mRNA levels [67]. *Helicobacter pylori*‐induced NAT10 can promote NAT10‐mediated *Mdm2* mRNA ac4C modification to stabilize *Mdm2* mRNA. This results in upregulated Mdm2 expression levels and increased p53 degradation, consequently impacting the occurrence and progression of GC [67]. Interestingly, NAT10 has also been reported to ubiquitinate Mdm2 and promote its degradation [8].

In addition, the long noncoding RNA (lncRNA) aspartyl‐tRNA synthetase antisense 1 (DARS‐AS1) can upregulate NAT10 expression by acting as a sponge for miR‐330‐3p. This mechanism leads to increased GC cell proliferation, migration, and invasion rates. A negative correlation has been observed between miR‐300‐3p and both DARS‐AS1 and NAT10 expression patterns [68]. NAT10 mediates *SEPT9* mRNA ac4C modification to promote its expression. SEPT9 further promotes nuclear accumulation of hypoxia‐inducible factor (HIF)‐1α in GC cells, which can activate genes involved in the homeostatic response to hypoxia. This results in glycolytic addiction formation, enhancing GC cell tolerance of the hypoxic environment. HIF‐1α can also regulate NAT10 gene transcription, thus suggesting that NAT10/SEPT9/HIF‐1α may form a positive feedback loop to influence glycolytic metabolism in GC cells [69].

### 4.3 CRC

Reduced glycogen synthase kinase‐3 activity leads to the subcellular redistribution of NAT10 in CRC cells, which enhances their motility [70]. Immunohistochemistry analysis showed that NAT10 protein expression levels were significantly increased in colon cancer cells and accumulated in the nucleus [71]. High NAT10 levels can promote CRC cell proliferation and predict shorter survival in CRC patients. In addition, NAT10‐mediated mRNA acetylation levels were increased in CRC and positively correlated with immune infiltration and microsatellite status in patients [72].

NAT10 can also modify ferroptosis suppressor protein 1 (*FSP1*) mRNA by ac4C in colon cancer cells, which positively affects *FSP1* mRNA stability and expression patterns. The inhibitory effect of FSP1 on ferroptosis may play an important role in the NAT10‐mediated promotion of colon cancer cell proliferation, migration, and invasion [71]. Moreover, miR‐6716‐5p can inhibit NAT10 expression, leading to reduced E‐cadherin levels. This contributes to the EMT of CRC cells and ultimately promotes their motility and invasion [42].

### 4.4 Bladder Cancer

NAT10 expression levels are abnormally elevated in bladder urothelial carcinoma (BLCA), which can predict poor patient prognosis. Knocking down NAT10 expression resulted in significantly reduced BLCA cell proliferation and invasion capabilities, as well as increased apoptosis. According to acRIP‐seq analysis, NAT10 can enhance the stability and translation efficiency of *BCL9L*, *SOX4*, and *AKT1* mRNAs by acetylating their wobble sites. These targets reportedly play important roles in a variety of tumor types [73, 74, 75] and displayed significantly decreased expression patterns following NAT10 knockdown. Therefore, NAT10 is believed to regulate the expression of these target genes by ac4C modification, thus promoting bladder cancer progression [76].

NAT10‐mediated ac4C modification has also been reported to be associated with cisplatin chemoresistance in bladder cancer. NAT10 can stimulate DNA damage repair by binding to and stabilizing *AHNAK* mRNA, thereby promoting bladder cancer cisplatin chemoresistance [3]. Therefore, inhibiting NAT10 potentially has value for enhancing cisplatin sensitivity.

### 4.5 BC

NAT10 expression levels are elevated in BC. NAT10 can acetylate MORC2 at K767, while SIRT2 can deacetylate MORC2. Acetylated MORC2 confers DNA damage‐induced H3T11 dephosphorylation and transcriptional repression of *CDK1* and *Cyclin B1*, thereby contributing to DNA damage‐induced G2 checkpoint activation. If MORC2 acetylation is defective, then the cells can pass the G2 checkpoint, thereby enhancing the sensitivity of the cell to DNA‐damaging agents. The MORC2 acetylation levels were positively correlated with NAT10 expression levels in human breast tumor samples. Therefore, NAT10‐mediated acetylation of MORC2 can modulate the DNA damage‐induced G2 checkpoint and is a potential therapeutic strategy for BC [17].

As previously described, NAT10 interacts with THUMPD1 to mediate tRNA ac4C modifications. THUMPD1 can reportedly promote BC cell migration and invasion by inhibiting E‐cadherin via the AKT‐GSK3β‐Snail pathway [77]. Moreover, treating BC cells with Remodelin, a NAT10 inhibitor, could reduce their resistance to doxorubicin by reversing the EMT [78].

### 4.6 Pancreatic Cancer

The LINC00623/NAT10 signaling pathway has been found to support pancreatic ductal adenocarcinoma (PDAC) progression. As a medium for NAT10 to interact with the deubiquitinase USP39, LINC00623 can bind to NAT10 and recruit USP39, thereby improving NAT10 stability by blocking its ubiquitination and degradation. Both LINC00623 and NAT10 were found to be highly expressed in PDAC tumor samples, with high NAT10 levels being significantly associated with poorer patient survival rates [79].

NAT10 can enhance oncogenic mRNA stability and translation efficiency by mediating mRNA ac4C modifications of mRNA, which supports PDAC development and progression. For example, NAT10 was found to promote PDAC progression by mediating *AXL* mRNA ac4C modification [80]. In addition, high NAT10 expression levels are associated with gemcitabine resistance in PDAC. Abnormal NAT10 expression patterns may also support angiogenesis in PDAC by activating the TGF‐β pathway, which contributes to distal tumor metastasis [57].

### 4.7 MM

Elevated NAT10 levels can promote MM cell proliferation. BCL‐XL, an antiapoptotic protein of the Bcl‐2 protein family, was found to be an important downstream target of NAT10 by acRIP‐seq analysis. NAT10 can enhance *BCL‐XL* expression by acetylation. Mechanistically, upregulated BCL‐XL protein can inhibit the downstream Bcl‐2‐associated X protein (BAX), an apoptotic protein, while upstream p‐AKT is increased to activate the PI3K‐AKT pathway. NAT10 can also activate the PI3K‐AKT pathway by upregulating CDK4 and CDK6 to accelerate MM cell proliferation [54]. The synergistic effect of the PI3K‐AKT pathway and Bcl‐2 protein is an important mechanism for inhibiting apoptosis and promoting tumor cell proliferation [81].

NAT10 can also increase centrosomal protein 170 (*CEP170*) mRNA acetylation levels, which improve its translation efficiency. Overexpressed CEP170 can promote MM cell proliferation and chromosomal instability [82]. Moreover, the NAT10 inhibitor Remodelin could inhibit MM cell growth.

### 4.8 AML

A case‐control analysis suggested that NAT10 expression levels were elevated in AML patients compared with healthy controls, with high NAT10 levels generally predicting poor survival [83]. Impaired apoptosis is one of the most significant signs of AML. Targeting NAT10 has been shown to inhibit AML cell proliferation and induce apoptosis, offering potential therapeutic benefits for treating this disease. Research has indicated that the expression levels of key cell cyclins (CDK2, CDK4, CyclinD1, and Cyclin E) decreased following downregulation of NAT10, while p16 and p21 expression levels were elevated. In addition, the expression levels of the apoptotic proteins Bax and Bak increased after NAT10 inhibitor treatment, while those of the antiapoptotic protein Bcl‐2 decreased. This led to the activation of the classical apoptotic pathway. Targeting NAT10 induced ERS and activated the unfolded protein response (UPR) pathway, ultimately leading to AML cell apoptosis [51]. Therefore, targeting NAT10 may be useful for AML treatment approaches by promoting cancer cell apoptosis.

### 4.9 Other Cancer Types

NAT10 inhibitors have been found to inhibit prostate cancer cell growth and proliferation, potentially because NAT10 can interact with CDC6 and bind to the DNA replication complex. In addition, targeting NAT10 is considered an effective method for castration‐resistant prostate cancer (CRPC) treatment [84].

In head and neck squamous cell carcinoma (HNSCC), high NAT10 expression levels can predict poor patient prognosis. When NAT10 expression was knocked down with siRNAs, HNCSS cell lines showed significantly increased cell cycle arrest in the G2/S phase. Additionally, NAT10 knockdown led to decreased cell proliferation, migration, and invasion rates [85].

## 5 NAT10 and Tumor Treatment

NAT10 expression is often associated with cancer drug resistance. In BLCA, NAT10 expression levels were elevated following cisplatin treatment and positively correlated with chemoresistance. Cisplatin induces p65 binding to the NAT10 gene promoter by activating the NF‐kB pathway, which in turn increases NAT10 transcription. NAT10 regulates *AHNAK* mRNA expression levels to enhance the DNA damage response, ultimately contributing to the development of cisplatin resistance in cancers [3]. In addition, NAT10 enhances doxorubicin resistance in HCC and BC by promoting the EMT [64, 78]. In HCC and BC cells, doxorubicin decreases E‐cadherin expression levels and enhances vimentin expression levels to promote the EMT, thus increasing tumor cell resistance to doxorubicin. Inhibiting NAT10 can reverse the doxorubicin‐induced EMT and decrease drug resistance.

Targeted inhibition of NAT10 has great potential value in cancer therapy. For example, using siRNAs to knock down NAT10 expression or Remodelin to inhibit NAT10 protein activity could reduce the HNSCC cell proliferation, migration, and invasion rates, significantly inhibiting tumor growth [85]. Remodelin can also enhance apoptosis, reverse both the EMT and drug resistance, and reduce FA accumulation in cancer cells [36]. Additionally, Remodelin can inhibit HIF expression, in turn affecting angiogenesis. The inhibitory effect of Remodelin on HIFs depends on the activity of NAT10 [86]. Remodelin could reduce glycolysis and mitochondrial damage in GC cells by inhibiting ac4C modification levels, effectively freeing the cancer cells from their dependence on glycolysis. A combination of Remodelin and antiangiogenic therapy could inhibit this cancer cell glycolytic addiction and reduce their tolerance to hypoxia, thus preventing their microenvironmental stress response and enhancing therapeutic efficacy [69]. NAT10 knockdown synergizes with PD‐L1 blockade therapy to enhance the immunotherapy efficacy of PD‐L1 blockade by reducing immunosuppression, improving immune surveillance, and inhibiting glycolysis in tumor cells [87]. Therefore, targeting NAT10 may be a reliable direction for future cancer treatment strategies.

## 6 Conclusions and Prospects

In this review, we systematically summarized the biological activities of NAT10 and its mechanisms of action in various cancers. NAT10 mediates the acetylation of numerous RNA and protein molecules, thereby regulating a variety of biological processes. Furthermore, NAT10 can impact tumor progression by targeting oncogenes and proteins, implicating NAT10 as a potential target for the diagnosis and treatment of a variety of cancer types.

**Figure 4:**
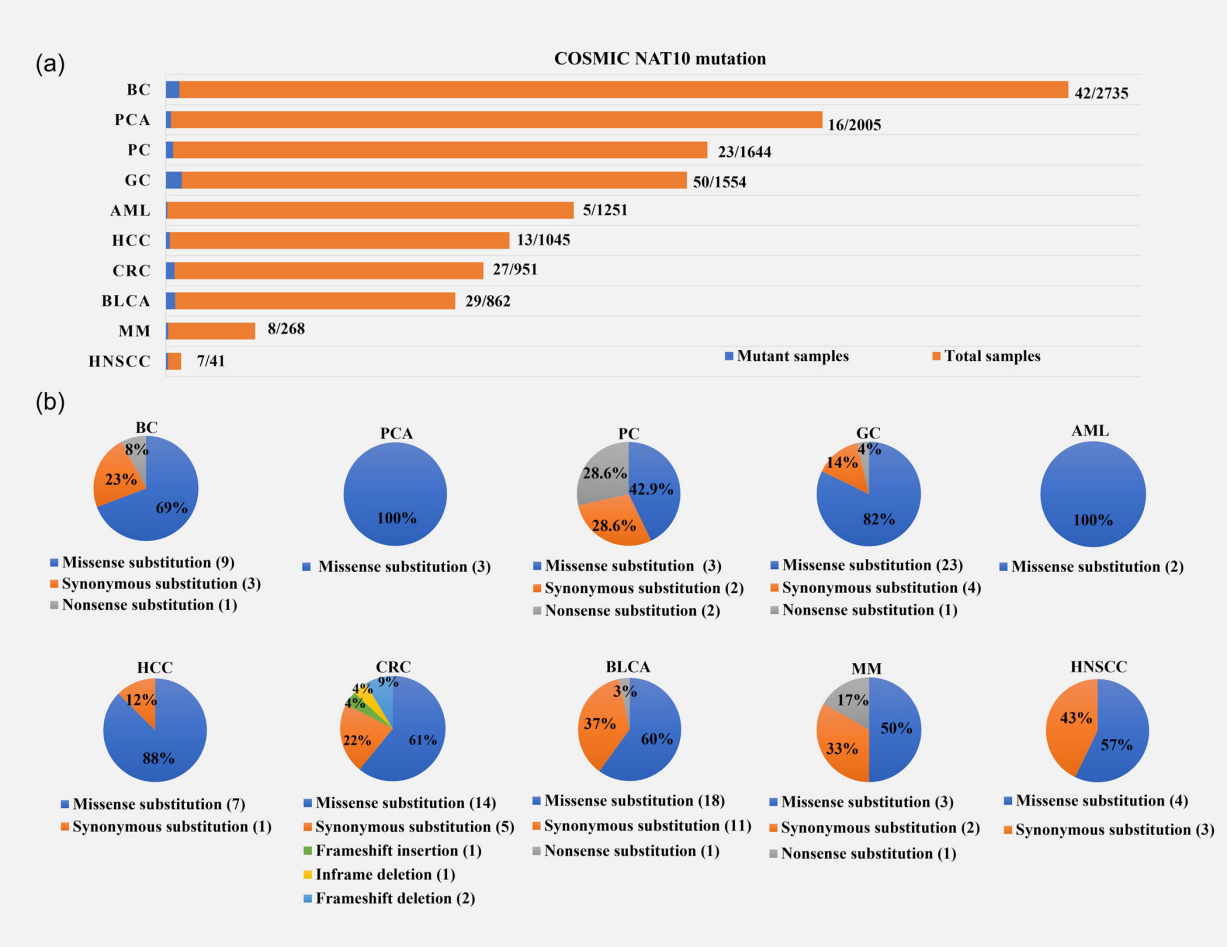
NAT10 mutations in cancer. (a) The proportion of NAT10 mutations in different cancer samples according to the COSMIC database. (b) Types of NAT10 mutations in different cancers. Numbers in parentheses indicate the number of samples. AML, acute myeloid leukemia; BC, breast cancer; BLCA, bladder urothelial carcinoma; CRC, colorectal cancer; GC, Gastric cancer; HCC, hepatocellular carcinoma; HNSCC head and neck squamous cell carcinoma; MM, multiple myeloma; NAT10, *N*‐acetyltransferase 10; PC, pancreatic cancer; PCA, prostatic cancer.

NAT10 is normally localized in the nucleus and involved in the cell cycle, ribosome formation, and DNA damage response. As the only known ac4C “writer” enzyme, NAT10 can regulate the mRNA stability and translation efficiency of multiple target genes through ac4C modification, thereby controlling their expression levels. Therefore, NAT10 can regulate the expression levels of target oncogenes via acetylation modification, which can ultimately affect tumor cell proliferation, metastasis, and invasion. In addition, NAT10 mediates the acetylation of proteins to participate in a variety of biological actions. For example, NAT10 acetylates tubulin and supports its stability, thus contributing to the normal progression of cell mitosis [6]. NAT10 can also affect cancer progression by acting on target proteins, such as p53 and MORC2. Recent studies have shown that NAT10 Khib modification at lysine 823 improves NAT10 protein stability and contributes to cancer metastasis [88]. However, further research on the effect of NAT10 on disease progression is needed. According to the COSMIC database, NAT10 is mutated in multiple cancer types (Figure 4). Genetic mutations can lead to dysregulated NAT10 expression patterns in cancer. Therefore, studying NAT10 mutation sites is potentially significant for increasing our understanding of cancer biology and exploring new methods for prevention and treatment.

High NAT10 expression levels often predict poor cancer patient prognosis. Inhibiting NAT10 reduces tumor cell drug resistance, which shows great potential for cancer therapy. Remodelin is the only currently known NAT10 inhibitor. It has been shown to inhibit cancer cell proliferation, migration, and drug resistance, although its clinical impact remains to be confirmed. Recent research has shown that Fosaprepitant, Leucal, Fludarabine, and Dantrolene may be potential novel inhibitors of NAT10, but these molecules require further verification [89].

In summary, we systematically outlined the biological characteristics of NAT10 and its effects on tumor proliferation, metastasis, and invasion. These results provide evidence for NAT10 as a potential biomarker for disease prognosis and therapy. NAT10‐mediated target acetylation may be a promising direction for disease treatment development. However, the value of NAT10 in such applications needs to be further confirmed with more clinical data. Identifying additional combination therapies associated with NAT10 knockdown is potentially valuable for future cancer treatment methods. Additionally, including Remodelin in more combination therapies will likely contribute to improved cancer patient outcomes. More NAT10 inhibitors need to be discovered and investigated. The development of NAT10 inhibitors with more selectivity and fewer side effects can be an important direction for future NAT10 research.

## Author Contributions

**Yufeng Han:** writing – original draft (equal), writing – review and editing (equal). **Xinxin Zhang:** funding acquisition (equal), writing – review and editing (equal). **Lei Miao:** writing – review and editing (equal). **Huiran Lin:** writing – review and editing (equal). **Zhenjian Zhuo:** funding acquisition (equal), supervision (equal), writing – review and editing (equal). **Jing He:** supervision (equal), writing – review and editing (equal). **Wen Fu:** supervision (equal), writing – review and editing (equal).

## Ethics Statement

The authors have nothing to report.

## Consent

The authors have nothing to report.

## Conflicts of Interest

Professor Zhenjian Zhuo is the member of the *Cancer Innovation* Editorial Board. To minimize bias, he was excluded from all editorial decision‐making related to the acceptance of this article for publication. The remaining authors declare no conflicts of interest.

## Data Availability

The authors have nothing to report.
